# Ultra-compact fiber tapering: plasmonics and structural bending as new combination of heat and pull

**DOI:** 10.1038/s41377-023-01203-5

**Published:** 2023-07-03

**Authors:** Tian Yang

**Affiliations:** State Key Laboratory of Advanced Optical Communication Systems and Networks, Key Laboratory for Thin Film and Microfabrication of the Ministry of Education, School of Electronic Information and Electrical Engineering, Shanghai Jiao Tong University, 200240 Shanghai, China

**Keywords:** Nanophotonics and plasmonics, Photonic devices

## Abstract

Fabrication of optical fiber tapers is realized with a combination of plasmonic microheaters and specially designed structural bending of optical fibers, which provide the necessary elements of “heat and pull”. The resultant compactness and flame-free condition enable monitoring of the tapering process inside a scanning electron microscope.

By tapering a standard optical fiber down to a total diameter on the wavelength scale, the fiber-guided lightwave would tunnel out of the silica body into the surrounding medium^1^. The evanescent field geometry of these micro- and nano-tapered fibers, together with their straightforward coupling to standard fiber-optic components, have facilitated sensing of light-matter interactions^2,3^, optical manipulation of matter^4^ and coupling between fiber-guided lightwaves and local optical resonators^5^ and emitters^6^.

The last two decades have witnessed a quickly increasing demand for micro- and nano-fibers in both scientific research and industry. At the same time, to achieve more flexible customized geometries and higher mechanical robustness, the fabrication techniques for these devices have been constantly upgrading and enriched in variety, the most widely used ones including direct drawing of silica nanowires^7^, the flame-brush method^8^ and CO_2_ laser fusion-splicing^9,10^. Current fabrication techniques require flames, high-power infrared lasers or arc discharges, all of which are mounted on bulky mechanical stages. In general, the key is to combine two indispensable elements: the heat source to soften the glassy materials and the pulling force to stretch the target fibers.

Now, writing in this issue of Light: Advanced Manufacturing^11^, Min Qiu’s team at Westlake University has thought outside the convention of the intuitive implementation of “heat and pull” (Fig. 1a). First, they replaced the external heating apparatus by a gold plate, which adhered to a pre-tapered fiber, so that plasmonic absorption assumed the role of heat source as the fiber-guided laser lightwave excited the plate plasmons through the evanescent optical field. Different from the team’s previous demonstration of the plate’s spiral motion under nanosecond-pulsed laser driving^12^, here they either increased the laser pulse repetition rate or used a continuous-wave laser to ensure that the local temperature at the heated region reached the softening point of the silica material (Fig. 1b). Second, they elaborately built a bending structure of the target fiber, the resultant inner stress of which assumes the role of pulling force. To do so, the fiber contained a transversely suspended region deformed in a prescribed door-shaped manner, the central segment of which was subject to tensile stress and was pulled when its silica material was heated and softened (Fig. 1c). Both the heating and pulling elements can be considered “internal”, given that the inputs are either propagating along the target fiber (photons) or embedded in its prescribed deformation pattern (stress). This unique feature makes the proposed method miniaturized and low power consumption, compared with previous fiber tapering techniques.

**Fig. 1:**
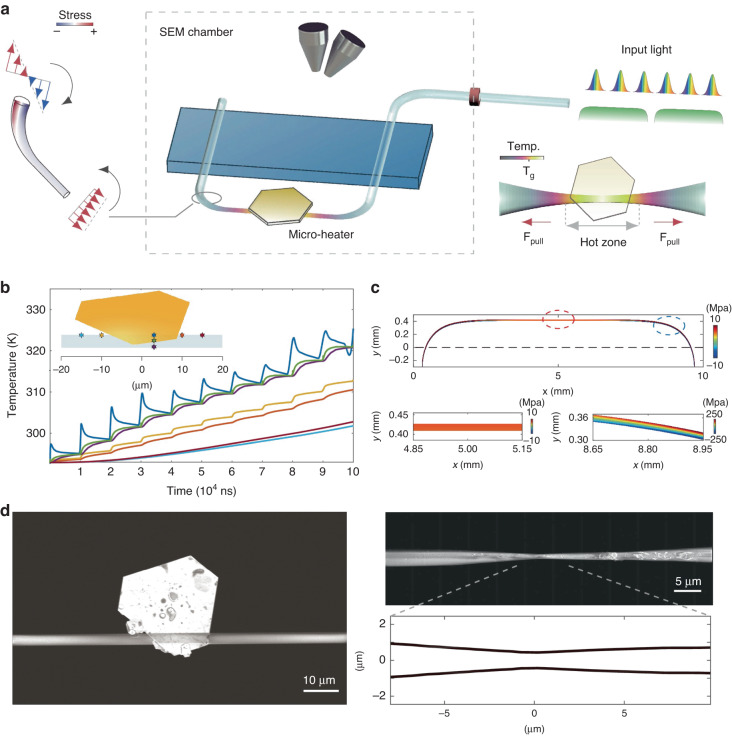
Fiber tapering using plasmonic microheaters and deformation-induced pull. **a** Schematic of the experimental set-up. **b** Simulated heat accumulation scheme at seven sampling points using pulsed light of 100 kHz repetition rate. Positions of the sampling points are indicated in the inset. **c** Distribution of the inner stress in a deformed fiber along the axial direction. Positive and negative values correspond to tensile and compressive stresses, respectively. **d** SEM images of the plate-fiber system before and after tapering

The compactness and vacuum compatibility of the proposed method allows SEM observation of the tapering process. As shown in Fig. 1d, the fabricated samples have a biconical geometry, which have a thinned waist with a sub-micrometer diameter and a few tens of micrometer length. The size of the plasmonically tapered region is significantly shorter than that of the samples prepared through conventional methods with the use of macroscale heaters, the latter being around millimeters or centimetres long. Monitoring and control of the dynamic tapering process via light source termination and resumption has also been demonstrated under SEM, which increases the controllability of the proposed method. We can imagine that such a miniaturized and dynamically controllable fiber micromachining technology will be valuable for making small footprint fiber-optic devices in situ^13,14^.

The immediate follow-up work would be to improve the reproducibility and programmability of the fabrication procedure. For example, the dimensions and profiles of both plasmonic heater and fiber stress pattern need further engineering. In addition, since the current method involves ablation of the gold plate during the heating process, the authors have shown that ablation and removal of gold plate wouldn’t affect the tapering capability. This phenomenon is worth further investigation to meet special fabrication requirements, for example, when the to-be tapered fiber is already embedded within an optical circuit. In the future, with the novel physics mechanisms for making micro- and nano-fibers such as what has been demonstrated in this work, and with the advancement of laser micromachining technology for glass^15,16^, we can expect more complex fiber taper structures with sub-wavelength feature sizes be made to achieve unprecedented functionalities.

## References

[1] Wu XQ, Tong LM (2013). Optical microfibers and nanofibers. Nanophotonics.

[2] Zhang L, Lou JY, Tong LM (2011). Micro/nanofiber optical sensors. Photon. Sens..

[3] Yu XC (2018). Optically sizing single atmospheric particulates with a 10-nm resolution using a strong evanescent field. Light Sci. Appl..

[4] Zhao XT (2020). Optical fiber tweezers: a versatile tool for optical trapping and manipulation. Micromachines.

[5] Armani DK (2003). Ultra-high-*Q* toroid microcavity on a chip. Nature.

[6] Jones R (2020). Collectively enhanced chiral photon emission from an atomic array near a nanofiber. Phys. Rev. Lett..

[7] Tong LM (2003). Subwavelength-diameter silica wires for low-loss optical wave guiding. Nature.

[8] Birks TA, Li YW (1992). The shape of fiber tapers. J. Lightwave Technol..

[9] Dimmick TE (1999). Carbon dioxide laser fabrication of fused-fiber couplers and tapers. Appl. Opt..

[10] Ward JM (2006). Heat-and-pull rig for fiber taper fabrication. Rev. Sci. Instrum..

[11] Jia QN (2023). Fibre tapering using plasmonic microheaters and deformation-induced pull. Light Adv. Manuf..

[12] Tang WW (2021). Micro-scale opto-thermo-mechanical actuation in the dry adhesive regime. Light Sci. Appl..

[13] Ghosh P (2018). Photothermal‐induced nanowelding of metal–semiconductor heterojunction in integrated nanowire units. Adv. Electron. Mater..

[14] Zhou N (2022). Strong mode coupling-enabled hybrid photon-Plasmon laser with a microfiber-coupled nanorod. Sci. Adv..

[15] Sundaram SK (2022). Glasses in a fraction of a second. Opt. Photon. N..

[16] Liu HG, Lin WX, Hong MH (2021). Hybrid laser precision engineering of transparent hard materials: challenges, solutions and applications. Light Sci. Appl..

